# Expansion and Precise CRISPR-Cas9 Gene Repair of Autologous T-Memory Stem Cells from Patients with T-Cell Immunodeficiencies

**DOI:** 10.21769/BioProtoc.5085

**Published:** 2024-10-20

**Authors:** Xun Li, Van Trung Chu, Christine Kocks, Klaus Rajewsky

**Affiliations:** 1 Immune Regulation and Cancer, Max-Delbrück-Center for Molecular Medicine in the Helmholtz Association (MDC), Berlin, Germany; 2 Genome Engineering and Disease Models, Max-Delbrück-Center for Molecular Medicine in the Helmholtz Association (MDC), Berlin, Germany

**Keywords:** Humans, Memory T cells, Immunodeficiencies, Gene therapy, Perforin, CRISPR-Cas9 systems, Ribonucleoproteins, Adeno-associated virus 6 (AAV6), Genome editing, Gene editing efficiency

## Abstract

The adoptive transfer of autologous, long-lived, gene-repaired T cells is a promising way to treat inherited T-cell immunodeficiencies. However, adoptive T-cell therapies require a large number of T cells to be manipulated and infused back into the patient. This poses a challenge in primary immunodeficiencies that manifest early in childhood and where only small volumes of blood samples may be available. Our protocol describes the ex vivo expansion of potentially long-lived human T memory stem cells (T_SCM_), starting from a limited number of peripheral blood mononuclear cells (PBMCs). Using the perforin gene as an example, we provide detailed instructions for precise gene repair of human T cells and the expansion of T_SCM_. The efficiency of precise gene repair can be increased by suppressing unintended non-homologous end-joining (NHEJ) events. Our protocol yields edited T-cell populations that are ready for phenotyping, genome-wide off-target analysis, and functional characterization.

Key features

• Expansion and enrichment of T_SCM_ from PBMCs using IL-7 and IL-15.

• Phenotyping of T_SCM_.

• Design of “off-the-shelf” gene-repair strategies based on knock-in of a single exon or complete cDNA and design of effective guide RNAs and DNA donor templates.

• High-efficiency gene targeting using CRISPR-Cas9, recombinant adeno-associated virus serotype 6 (rAAV6), and a selective small molecule inhibitor of DNA-dependent protein kinase (DNA-PK).

## Graphical overview


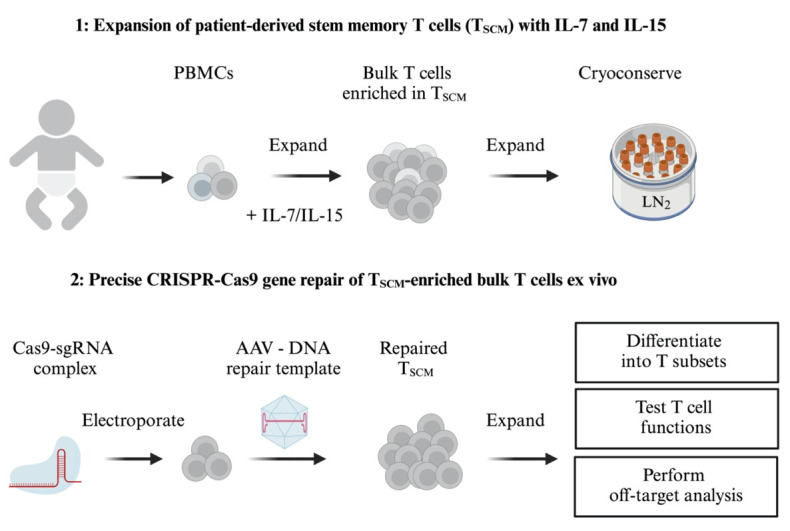


## Background

Familial hemophagocytic lymphohistiocytosis (FHL) is a group of inherited, potentially fatal inflammatory diseases caused by loss-of-function mutations in the NK-cell and T-cell cytotoxic machinery. Patients with FHL2 carry mutations in the perforin-1 gene (*PRF1*). This protocol is an extension of our previous work with primary, perforin-deficient T cells from a genetic mouse model that mimics FHL2 [1,2]. We isolated memory T cells from these mice, repaired their perforin genes, and showed that treatment with these repaired T cells was able to overcome lethal hyperinflammation [2]. The repaired T cells efficiently killed EBV-triggered malignant B cells in vivo, thereby removing the inflammatory stimulus and restoring immune homeostasis [2].

In the same study, we also showed that human T cells can be repaired in a similar way, setting the stage for translation to the clinic [2]. Ideally, T-cell therapy is performed with antigen-experienced memory T cells that are able to differentiate in vivo into different effector subsets, are long-lived, and possess self-renewing capacities [3]. T memory stem cells (T_SCM_) reside in CD8^+^ and CD4^+^ T-cell precursor subsets that express CD62L [4]. For the CD8 T-cell compartment, it has been shown that CD62L^+ ^cells possess the capacity for immune reconstitution, self-renewal, and persistence in vivo [5]. A combination of recombinant IL-7 and IL-15 can be used to generate such T_SCM_ by activating and expanding a pool of precursor T cells present in blood [6–8]. IL-7 is required for the development of these cells, IL-15 maintains their proliferation, and their gene expression signature resembles naturally occurring T_SCM_ [6].

This protocol describes a highly efficient gene repair strategy for human primary T cells, starting from the typically limited number of peripheral blood mononuclear cells (PBMCs) that can be obtained from critically ill, immunodeficient children in a clinical setting. Ex vivo expansion and enrichment of potentially long-lived T_SCM_ cells is followed by nucleofection of ribonucleoprotein complexes (RNPs) [9] composed of guide RNA (gRNA) and Cas9 nuclease protein to introduce site-specific DNA double-strand breaks. The T cells are then infected with recombinant adenovirus-associated virus serotype 6 (rAAV6) to deliver the DNA template for host cell–mediated homology-directed repair (HDR) [10] and are treated with a small molecule non-homologous end-joining (NHEJ) inhibitor to increase HDR efficiency [8,11]. This strategy achieves highly efficient gene repair in T cells from patients with FHL, resulting in CD8^+^ T-cell populations carrying more than 85% mCherry reporter knock-ins (see Suppl. Figure S2 in Li et al. [2]).

For effective gene repair, it is desirable to develop “off-the-shelf” strategies that repair as many known disease mutations as possible. We illustrate this principle using the human *PRF1* locus, whose exons 2 and 3 encode a 555 amino acid polypeptide [12,13]. By replacing a mutated exon 3 with a corrected version, approximately two-thirds of ~60 known *PRF1* disease mutations can be repaired [13]. By replacing exon 3 with the full-length coding region of *PRF1*, 20 additional disease mutations in exon 2 can be repaired. In both cases, the genetic makeup of the repaired T cells is only minimally altered, and the repaired *PRF1* gene is expressed under its physiological transcription and translation control elements.

PBMC samples from FHL2 patients are difficult to obtain and therefore precious, and contain only a limited number of cells. Therefore, we also provide a protocol for cryopreservation of expanded patient T_SCM_ cells. Our protocol does not require sorting steps for T-cell isolation and can be performed repeatedly if needed, allowing optimization of gene editing conditions, scaling up of T-cell culture, and potentially multiple rounds of patient treatment. Expanded patient T cells showed no signs of T-cell exhaustion [14–16], neither before nor after gene editing.

## Materials and reagents

**Biological materials**

1. Peripheral blood mononuclear cells (PBMCs) from FHL patients and healthy donors (controls)

**Reagents**
1. OPTIONAL, see step A1 note: Addgene plasmid #64324 [pU6-(BbsI) CBh-Cas9-T2A-mCherry]
2. OPTIONAL, see step A1 and A2 notes: Addgene plasmid #86457 [MSCV-hU6-(BbsI)-CcdB-(BbsI)-PGK-Puro-T2A-BFP]
3. FuGENE^®^ HD transfection reagent (Promega, catalog number: E2311)
4. Opti-MEM^TM ^serum-reduced medium (Fisher Scientific, catalog number: 31985062)
5. QuickExtract^TM ^DNA extraction solution (Lucigen, catalog number: QE0905T)
6. Alt-R^®^ CRISPR-Cas9 crRNA 2 nmol [Integrated DNA Technologies (IDT)] (see Table 1 and Figure S1)
  **Table 1.** Guide RNA (gRNA) sequences for editing the coding part of exon 3 of the *PRF1* gene [gene ID 5551; *Homo sapiens* chromosome 10, GRCh38.14, NC_000010.11(70597348..70602741, complement]. Only the 20 target-specific nucleotides are shown in the third column (see also Figure S1). The preferred protospacer-adjacent motif (PAM) for Cas 9 is NGG, shown in bold and underlined.

Species	Name	Sequence (5′→3′)	Genome_coordinates[strand]
Human	gPRF1.1	GCGGGGGAGTGTGTACCACA **TGG**	Chr10:70599156:70599178[+]
Human	gPRF1.2	GGAGCTGGGTGGCCGCATAT **CGG**	Chr10:70599018:70599040[-]
7. Alt-R^®^ CRISPR-Cas9 tracrRNA 100 nmol (IDT, catalog number: 1072534) (see Figure S1)
8. Alt-R^TM^ *S.p.* Cas9 nuclease V3 or Alt-R^TM^ *S.p.* HiFi Cas9 nuclease V3 (IDT, catalog number: 1081059 or 1081061)
9. De novo synthesized codon-modified DNA repair template (GeneArt Gene Synthesis Services, Thermo Fisher Scientific)
10. pAAV-GFP Control Vector (Cell BioLabs, catalog number: VPK-400)
11. NEBuilder^®^ HiFi DNA Assembly Cloning Kit (New England BioLabs, catalog number: E5520S)
12. One Shot^TM^ Stbl3^TM^ chemically competent *E. coli* ^TM ^(Thermo Fisher Scientific, catalog number: C737303)
13. ImmunoCult^TM^-XF T cell expansion medium (STEMCELL Technologies, catalog number: 10981)
14. Human recombinant IL-7 (5 ng/mL) (PeproTech, catalog number: AF-200-07)
15. Human recombinant IL-15 (5 ng/mL) (PeproTech, catalog number: AF-200-15)
16. ImmunoCult^TM^ human CD3/CD28/CD2 T-cell activator (STEMCELL Technologies, catalog number: 10970)
17. CryoStor^®^ CS10 cryopreservation medium with 10% DMSO (STEMCELL Technologies, catalog number: 07930)
18. Phosphate-buffered saline (PBS) pH 7.2, without calcium/magnesium (Life Technologies, catalog number: 20012-068)
19. Bovine serum albumin fraction V (BSA) (Carl Roth, catalog number: 8076.3)
20. EDTA (Ethylendiaminetetraacetic acid disodium salt) solution pH 8 (0.5 M) (Sigma-Aldrich, catalog number: 03690)
21. LIVE/DEAD^TM^ Fixable Near IR (876) Viability Kit (Thermo Fisher Scientific, catalog number: L34981)
22. Human recombinant IL-2 (25 ng/mL) (PeproTech, catalog number: 200-02)
23. P3 Primary Cell 4D-Nucleofector^TM^ X kit L (Lonza, catalog number: V4XP-3012)
24. Dimethyl sulfoxide (DMSO) 10 mL (Sigma-Aldrich, catalog number: D2438)
25. M3814 (Nedisertib; DNA-PK inhibitor; CAS number: 1637542-33-6) (MedChemExpress, catalog number: HY-101570)
26. Ethanol absolute without additives, BioUltra 500 mL (Sigma-Aldrich, catalog number: 51976)
27. Phorbol-12-myristate-13-acetate (PMA) 1 mg (Sigma-Aldrich, catalog number: P8139)
28. Ionomycin calcium salt from *S. conglobatus* 1 mg (Sigma-Aldrich, catalog number: I0634)
29. Brefeldin A solution (1,000×) (BioLegend, catalog number: 420601)
30. BD Cytofix/Cytoperm^TM^ Fixation/Permeabilization Kit (BD Biosciences, catalog number: 554714)
31. QuickExtract^TM^ DNA extraction solution (LGC Biosearch Technologies/Lucigen, catalog number: QE09050)
32. PrimeSTAR^®^ GXL DNA polymerase (TaKaRa, catalog number: R050B)
33. CloneJET PCR cloning kit (Thermo Fisher Scientific, catalog number: K1231)
34. Antibodies:
  - Brilliant Violet 785^TM^ anti-human CD8, clone SKI (BioLegend, catalog number: 344740)
  - APC anti-human CD62L, clone DREG-56 (BioLegend, catalog number: 304810)
  - PE/Cyanine7 anti-human CD45RA, clone HI-100 (BioLegend, catalog number: 304126)
  - FITC anti-human CD95 (Fas), clone DX2 (BioLegend, catalog number: 305606)
  - Brilliant Violet 421^TM^ anti-human CD279 (PD-1), clone NAT105 (BioLegend, catalog number: 367422)
  - PE/Dazzle^ TM^ 594 anti-human TIGIT (VSTM3), clone A15153G (BioLegend, catalog number: 372716)
  - PE anti-human Perforin, clone B-D48 (BioLegend, catalog number: 353304)
  - Optional, see step C15: PE anti-human CD366 (Tim-3), clone F38-2E2 (BioLegend, catalog number: 345006)
  - Optional, see step C15: Brilliant Violet 711^TM^ anti-human CD223 (LAG-3), clone 11C3C65 (BioLegend, catalog number: 369319)
  - Optional, see step C15: APC/Cyanine7 anti-human CD39, clone A1 (BioLegend, catalog number: 328226)

**Solutions**

1. Bovine serum albumin (BSA) stock solution (10%) (see Recipes)
2. MACS buffer (see Recipes)
3. P3 electroporation buffer (see Recipes)
4. M3814 (Nedisertib) stock solution (25 mM) (see Recipes)
5. Phorbol myristate acetate (PMA) stock solution (50 ng/mL) (see Recipes)
6. Ionomycin stock solution (500 ng/mL) (see Recipes)

**Recipes**

1. **Bovine serum albumin (BSA) stock solution (10%)**
  Layer 10 g of BSA over 100 mL of PBS in a glass beaker and let it dissolve overnight at 4 °C. Sterile-filter through a 0.2 µm PES bottle-top filter. Store at 4 °C or freeze in aliquots at -20 °C.
2. **MACS buffer (PBS pH 7.2 containing 0.5% BSA and 2 mM EDTA)**
  To 500 mL of PBS pH 7.2 (without calcium and magnesium), add 25 mL of sterile 10% BSA stock solution and 2 mL of 0.5 M EDTA solution pH 8. Sterile-filter through a 0.2 µm PES bottle-top filter.
**CRITICAL:** LIVE/DEAD^TM^ fixable stains are only compatible with flow cytometry buffers containing less than 1% of protein.
3. **P3 electroporation buffer**
  Just before use, freshly prepare 20 µL of P3 electroporation buffer by adding 3.6 µL of Supplement 1 to 16.4 µL of P3 primary cell Nucleofector^TM^ solution (P3 Primary Cell 4D-Nucleofector^TM^ X kit, Lonza).
4. **M3814 (Nedisertib) stock solution (25 mM)**
  Dissolve 5 mg of M3814 in 415 µL of DMSO (25 mM = 5,000×). Store in 10 µL aliquots at -80 °C. Dilute the stock solution 1:100 in PBS pH 7.2 to make a 50× working stock solution (250 µM = 50×). Aliquot the working stock solution and store at -20 °C for up to 4 weeks.
5. **Phorbol myristate acetate (PMA) stock solution (50 ng/mL)**
  Dissolve 1 mg of PMA in 20 mL of ethanol (50 ng/mL final concentration). Store in 10 µL aliquots at -20 °C.
**CAUTION:** Phorbol ester is a tumor promoter. Wear personal protective equipment including gloves.
6. **Ionomycin stock solution (500 ng/mL)**
  Dissolve 1 mg of ionomycin calcium salt in 2 mL of ethanol (500 ng/mL final concentration). Store in 10 µL aliquots at -20 °C.

**Laboratory supplies**

1. Tissue culture 96-well plates, flat bottom (Sarstedt, catalog number: 83.3924)
2. Tissue culture 96-well plates, U bottom (Sarstedt, catalog number: 83.3926.500)
3. Tissue culture 48-well plates (Sarstedt, catalog number: 83.3923)
4. Tissue culture 24-well plates (Sarstedt, catalog number: 83.3922)
5. Tissue culture 12-well plates (Sarstedt, catalog number: 83.3921)
6. Tissue culture 6-well plates (Sarstedt, catalog number: 83.3920)
7. Tissue culture dish 100 × 20 mm (Sarstedt, catalog number: 83.3902)
8. PIPETMAN^®^ single-channel pipettes P10, P20, P200, P1000 (Gilson)
9. Pipette filter tips [Sarstedt, catalog numbers: 70.3010.275 (10 µL), 70.3030.265 (20 µL), 70.3030.375 (100 µL), 70.3030.110 (200 µL), 70.3060.275 (1,000 µL)]
10. 0.2 mL PCR tubes with domed lids, 8-well strips (neoLab, catalog number: 7-5207)
11. 1.5 mL tubes (Eppendorf, catalog number: 0030 120.086)
12. 2.0 mL tubes (Eppendorf, catalog number: 0030 120.094)
13. 15 mL conical centrifugation tubes Falcon^TM^ 352097 (Thermo Fisher, catalog number: 10136120)
14. Mr. Frosty^TM^ freezing container (Thermo Fisher, catalog number: 5100-0036)
15. 2 mL CryoPure tubes (freezing vials) (Sarstedt, catalog number: 72.380.992)
16. Disposable PES filter units 0.2 µm, 150 mL (Thermo Scientific Nalgene, catalog number: 596-3320)
17. Disposable PES filter units 0.2 µm, 500 mL (Thermo Scientific Nalgene, catalog number: 595-3320)
18. Standard 96-well microtiter plate, U bottom (Thermo Scientific Abgene, catalog number: AB0796)

## Equipment

1. Tissue culture incubator set at 37 °C, 5% CO_2_
2. Refrigerated centrifuge with adapters for plates and 15 mL conical tubes (e.g., Eppendorf Centrifuge 5810R)
3. Benchtop centrifuge for 1.5 mL/2 mL tubes (e.g., Eppendorf Centrifuge 5424)
4. BD LSRFortessa cell analyzer flow cytometer (BD Bioscience)
5. TC20^TM^ automated cell counter (Bio-Rad, catalog number: 1450102)
6. 4D-Nucleofector^®^ X Unit (Lonza, catalog number: AAF-1003B)
7. Bright-Line^TM^ hematocytometer (Sigma-Aldrich, catalog number: Z359629)
8. Optional; see steps A2 and A3: BD FACSAria III Cell Sorter (BD Biosciences)

## Software and datasets

1. CrispRGold (free) (https://crisprgold.mdc-berlin.de/) or CRISPOR (free) (http://crispor.tefor.net/)
2. ICE CRISPR Analysis Tool (Synthego) (free)
https://www.synthego.com/products/bioinformatics/crispr-analysis
3. GeneOptimizer Algorithm (GeneArt Gene Synthesis Services, Thermo Fisher Scientific) (free)
https://www.thermofisher.com/de/de/home/life-science/cloning/gene-synthesis/geneoptimizer.htmL
4. FlowJo (v.10, FlowJo, BD Biosciences) (commercial analysis software, requires a license)
5. ApE (A Plasmid Editor) (free) (https://jorgensen.biology.utah.edu/wayned/ape/)

## Procedure

- **Design and experimental assessment of gRNA efficiency**
*Note: gRNAs contain the target-specific sequence for guiding Cas9 protein to a genomic location. We use a two-part guide RNA annealed to form an overlapping duplex (Figure S1). One part of this two-part gRNA consists of a non-variable 67-nucleotide-long tracrRNA; the other consists of a partly variable 36-nucleotide-long crRNA that harbors 20 target-specific nucleotides. Alternatively, you can use a format that combines these RNAs in a 100-nucleotide-long single-guide RNA. We prefer the former option because it reduces costs.* ***See General note 1***.
  1. Design gRNAs with CrispRGold (https://crisprgold.mdc-berlin.de/) [17] around the selected genomic target site [coding part of exon 3 of the *PRF1* gene (Gene ID 5551; *Homo sapiens* chromosome 10, GRCh38.14, NC_000010.11[70597348..70602741, complement)]. Alternatively, gRNAs can be designed with CRISPOR (http://crispor.tefor.net/) [18]. The software outputs the 20 target-specific nucleotides in the gRNA. For examples of gRNAs targeting exon 3 of the *PRF1* gene, see Table 1, Figure 1, and Figures S2–S4.
*Note: To reduce costs, we often pre-select efficient gRNAs by first ordering oligo duplexes, cloning them into mammalian expression vectors, and transfecting them into cells [19]. Alternatively, to save time, you can skip step A2 and order several crRNAs right away from Integrated DNA Technologies (IDT), assemble the corresponding RNPs (steps D1–D5), electroporate them into activated primary human T cells (steps F1–F12), and then proceed as described in steps A3–A5 below.*
  2. Order forward and reverse DNA oligos [with cloning-compatible overhangs as configured by CrisprGold (Figure S3) or CRISPOR] corresponding to the 5–6 most high-ranking gRNAs (specificity score ≥ 12 in CrispRGold; Figure S3), phosphorylate, anneal, and clone them via *BbsI* sites (Figure S5) into a guide RNA/Cas9 expression plasmid, and transfect into HEK293T cells. We use the plasmid pU6-(BbsI) CBh-Cas9-T2A-mCherry (Addgene plasmid #64324, https://www.addgene.org/64324/; Chu et al. [20]). It was modified by us to express the fluorescent marker mCherry and is originally based on Addgene plasmid #42230, https://www.addgene.org/42230/; Cong et al. [21], Ran et al. [22]). For transfection, we use 5 × 10^5^ HEK293T cells in a 6-well plate and transfect them with 9 µL of FUGENE^®^ HD transfection reagent with 3 µg of sgRNA-expression plasmid (ratio 3:1) completed with Opti-MEM^TM^ to a final volume of 150 µL. We routinely obtain a transfection efficiency of >80%.
*Note: In order to be able to select for transfected cells by fluorescence-activated cell sorting (FACS), we often use the expression plasmid MSCV-hU6-(BbsI)-CcdB-(BbsI)-PGK-Puro-T2A-BFP carrying a blue fluorescent protein (BFP) reporter (Addgene Plasmid #86457*, *https://www.addgene.org/86457/* *; Chu et al. [19]) and transfect into Cas9-expressing suspension cells, such as BJAB lymphoma cells by electroporation. (Any human Cas9-expressing cell line can be used for this purpose. A large selection of Cas9-expressing cell lines is commercially available.)*
  3. Three days after transfection, isolate genomic DNA from the transfected cells [along with unedited (non-transfected) control cells] (50 µL of QuickExtract^TM^ DNA Extraction Solution per 10^5 ^cells) and PCR-amplify a 500–1,000-bp region around the targeted genome site. Send the PCR amplicons to Sanger sequencing.
*Note: Alternatively, one day after transfection, first isolate BFP-expressing (transfected) cells by FACS and expand the sorted cells for two days, before isolating genomic DNA. gRNA action through Cas9 RNP delivery reaches a maximum after approximately 24 h (Kim et al. [23], see Figure 6B).*
  4. Assess the genome editing efficiency of your guide RNAs by *Inference of CRISPR Edits* (ICE) [24]. Upload Sanger trace data (.ab1 files) to the free ICE CRISPR Analysis Tool (https://www.synthego.com/products/bioinformatics/crispr-analysis) (Figure S6).
**CRITICAL:** High-quality Sanger sequencing data are required for this step. **See also General note 1.**
  5. Order the two highest-scoring crRNAs, as well as tracrRNA, and *S.p.* Cas9 or *S.p.* HiFi Cas9 from a commercial supplier such as IDT. (For applications where high fidelity of gene repair is critical, we recommend HiFi Cas9.)
**CRITICAL:** Good gRNAs are extremely important for achieving efficient and reliable gene editing. The higher the predicted knockout efficiency, the better. Generally, we aim for an ICE indel percentage of 60% or higher (Figure S6).
- **Design of DNA donor templates**
  Gene correction strategy examples for the PRF1 gene are shown in Figure 1.
  **Figure 1.**
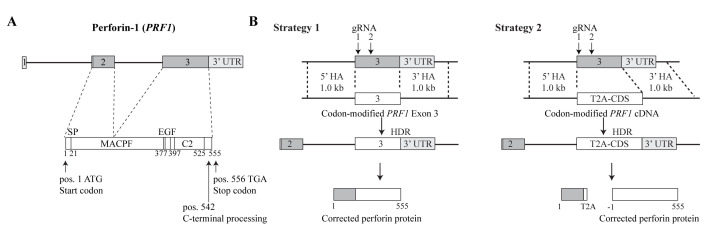
Examples of two gene correction strategies for the *PRF1* gene. A. Schematic representation of the human *PRF1* gene and the encoded 555 amino acid perforin polypeptide. UTR, untranslated region; SP, signal peptide (cleaved off after translocation of the nascent protein into the ER); MACPF, membrane attack complex/perforin domain (includes the central machinery of pore formation); EGF, EGF-like domain (forms a shelf-like assembly connecting MACPF and C2 domain); C2, C2 domain (calcium-dependent phospholipid binding). B. Two gene correction strategies. With both strategies, the native exon 3 coding part gets replaced by a repaired version whose sequence has been diversified (codon-modified, see Procedure step B2). Repair templates are codon-modified to prevent unwanted HDR events that do not lead to gene repair. Strategy 1 replaces the mutated version of exon 3 with a repaired version. For a more comprehensive gene correction by cDNA knock-in [25], strategy 2 replaces exon 3 with the full-length *PRF1* coding sequence (CDS): In place of the 5′ UTR, we engineered the cDNA to start with a T2A “self-cleaving peptide” preceded by a flexible serine-glycine-linker (TCC.GGC.AGC.GGC) [26], that is followed by the *PRF1* ATG start codon, the signal peptide, and the complete *PRF1* coding sequence ending with the TGA stop codon. Strategy 2 places the *PRF1* CDS under endogenous transcriptional and translational control and allows correction of the ~60 known pathogenic *PRF1* mutations (with the exception of frameshift or nonsense mutations in exon 2) [12,13,27]. Cleavage sites for the two guide RNAs (gRNA) are indicated. We use two gRNAs for gene repair in primary T cells because this increases the gene repair efficiency (see Li et al. [2] Suppl. Table S1). HA, homology arm; HDR; homology-directed repair. Adapted from Figure 6 in Li et al. [2].
  1. Design a DNA donor template carrying 5′ and 3′ homology arms of at least 0.5–1.5 kb upstream and downstream of the coding part of exon 3 of *PRF1* (Figure 1). Design primers and PCR-amplify homology arms from the genomic DNA from a healthy donor, incorporating 20–30 bases overlap to exon 3 (or the T2A-cDNA) and the *NotI*-linearized backbone vector (Figure S7). These overlaps are required for the “one-step cloning” as described below in step B4.
*Note: Generally, we aim for a minimum length of 500 bp but up to 1 kb or more if the packaging capacity of the adeno-associated virus (AAV) vector allows it (maximally 4–4.4 kb insert size excluding inverted terminal repeats;* *https://www.vectorbiolabs.com/faq-aav/* *). See also Figure S7.*
  2. Modify the nucleotide sequence of the replaced exon or the cDNA in the DNA repair template by using the free Gene Optimizer Algorithm (GeneArt Gene Synthesis Services, Thermo Fisher Scientific) [28]. We refer to this modified exon 3 repair template as “codon-modified” (see Figure 1).
**CRITICAL:** This step is intended to retain high expression of the repaired gene while diversifying the nucleotide sequence to prevent unwanted, small HDR events that would not lead to gene repair. These can occur in DNA stretches with extended homologies between the repair template and the native genomic locus.
  3. Order a de novo synthesized DNA fragment corresponding to the DNA repair template.
  4. Assemble the whole HDR template for “one-step cloning” into the AAV vector: Mix NEBuilder^®^ HiFi DNA Assembly Master Mix with 0.5 pmole each of the 5′ and 3′ homology arms, the codon-modified exon 3 (or T2A-cDNA), and the pAAV-GFP backbone vector (linearized with *NotI*) (Figure S7), and incubate the reaction at 50 °C for 60 min.
  5. Transform into chemically competent *E. coli* such as the HB101-derived strain Stbl3, which supports cloning of inserts containing repetitive elements, and purify the plasmids containing the HDR repair templates. (Owing to the high efficiency of NEBuilder^®^ HiFi DNA Assembly, we usually obtain approximately 30% of colonies with inserts at this step.)
*Note: Alternatively, you can assemble the HDR template by one-step cloning into a NotI linearized intermediate cloning vector, and only clone the assembled NotI fragment into pAAV in a second step. We also deposited cloning plasmids containing the assembled HDR templates for PRF1 targeting (NotI-fragment) (Addgene plasmids # 209075 and 209076).*
  6. Confirm the sequence of the HDR repair templates by Sanger sequencing.
**CRITICAL:** The viral vector carries an inverted terminal repeat (ITR) at each end. These sequences are repetitive and make cloning difficult and the pAAV plasmids unstable. Since this can affect AAV production and targeting efficiency downstream, we recommend submitting the plasmid to full plasmid next-generation sequencing to a commercial supplier such as Genewiz or Eurofins Genomics LLC.
- **Expansion and enrichment of T memory stem cells from peripheral blood mononuclear cells (PBMCs) using IL-7 and IL-15**
  A general workflow for the expansion of T memory stem cells is shown in Figure 2.
  1. Thaw a vial of frozen PBMCs (from a patient with FHL2 or a healthy donor) and transfer cells to 5 mL of prewarmed Immunocult XF T-cell expansion medium in a 15 mL conical tube. Isolation and cryopreservation of PBMCs is a routine procedure in clinical settings; see Puleo et al. [29] for more details.
  2. Activate bulk T cells (T cells containing all subsets): Pellet cells at 300× *g* for 5 min at 4 °C, gently remove supernatant, and resuspend the cell pellet in ImmunoCult^TM^-XF T cell expansion medium with human IL-7 (5 ng/mL) and IL-15 (5 ng/mL) in the presence of ImmunoCult^TM^ human CD3/CD28/CD2 T-cell activator (25 μL/mL) at a density of 1 × 10^6^/mL in a well of a 12- or 6-well plate (for up to 2 or 4 mL, respectively). Incubate cells at 37 °C and 5% CO_2_ for approximately 72 h.
  3. Collect activated bulk T cells and pellet cells at 300× *g* for 5 min at 4 °C, gently removing the supernatant.
  4. Resuspend cells with fresh ImmunoCult^TM^-XF T cell expansion medium with human IL-7 (5 ng/mL) and IL-15 (5 ng/mL) at a density of approximately 0.5 × 10^6 ^cells/mL in wells of a 6-well plate (up to 4 mL) or 100 mm culture dishes (up to 10 mL).
  5. Incubate for another 5–7 days. During this period, change the medium and split cells to a density of approximately 0.5 × 10^6^/mL every 2–3 days, as needed. We recommend a maximum density of approximately 1 × 10^6^ cells per milliliter. Approximately 95% of the cells are CD3^+^ T cells at this point, half of which are CD8^+^ T cells that have expanded 600–700-fold, as estimated from two independent experiments. Within the CD8^+^ T-cell compartment, most of the cells were T_SCM_ cells; see step C15 and Figure 3.
    **Figure 2.**
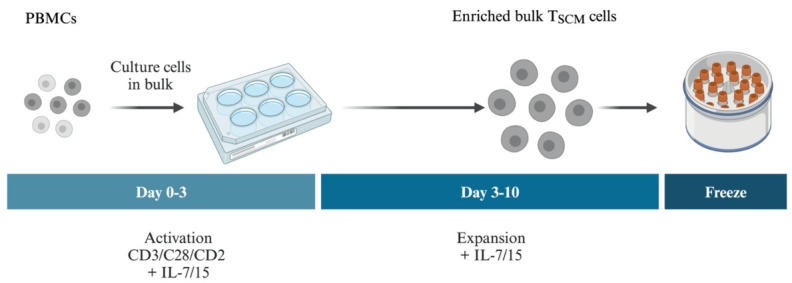
Workflow for expanding and enriching T memory stem cells (T_SCM_) from peripheral blood mononuclear cells (PBMCs). Starting from frozen aliquots of PBMCs, cells are cultured in bulk and the T cells (including all T-cell subsets) are selectively activated in the presence of IL-7 and IL-15 for 3 days, then further expanded in the presence of IL-7 and IL-15. IL-7 is required for the development of T_SCM_ and IL-15 maintains their proliferation without promoting their differentiation into effector subsets [6]. Cells are then frozen down for later use.
    **Figure 3.**
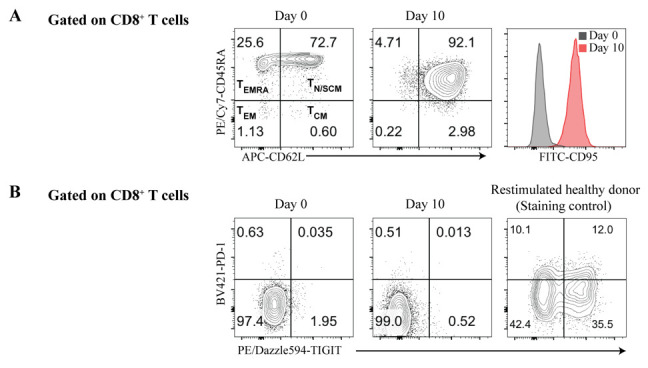
Phenotype of CD8^+^ T memory stem cells (T_SCM_) from a patient with familial hemophagocytic lymphohistiocytosis (FHL) after selective expansion and enrichment from peripheral blood monocytic cells (PBMCs). A. Flow cytometry analysis of CD8^+^ T cells before (Day 0) and after expansion (Day 10) from PBMCs isolated from a FHL patient. CD8^+ ^T cells expanded 600–700-fold, starting from 10^4 ^to 4 × 10^4^ cells (n = 2). Expanded CD8^+^ T cells stained positive for the naïve T-cell markers CD45RA and CD62L, but were almost exclusively CD95^+^, distinguishing them from CD45R^+^CD62L^+^CD95- naïve T cells and identifying them as T_SCM_-like. B. The expanded CD8^+^ T cells did not express the T-cell exhaustion markers PD-1 and TIGIT compared with the control staining. Thus, expanded CD8^+^ T cells from FHL patients showed no signs of T-cell exhaustion, an important prerequisite for T-cell therapy. T_EMRA_, effector memory T cells re-expressing CD45RA; T_EM_, effector memory T cells; T_N_, naïve T cells; T_SCM_, T memory stem cells; T_CM_, central memory T cells.
  6. Prepare the expanded patient T cells for cryo storage: Reserve some cells for flow cytometry analysis (approximately 2–5 × 10^5^ cells in a 96-well U-bottom plate; enough for two to maximum five staining reactions), pellet the remaining cells at 300× *g* for 5 min at 4 °C, gently remove supernatant, resuspend the cell pellet in ice-cold CryoStor^®^ CS10 solution, and transfer the cell suspension to cryovials (5 × 10^5^ to 1 × 10^6^ cells in 0.5 mL per vial).
  7. Cryopreserve cells using a Mr. Frosty^TM^ freezing container, store them at -80 °C overnight, and transfer them to a liquid nitrogen tank on the next day.
  8. Prepare the cells for flow cytometry analysis: Wash the reserved cells (from step C6) once with 100 μL of MACS buffer and pellet at 300× *g* for 5 min at 4 °C; then, carefully remove the supernatant without disturbing the cell pellet.
  9. Reconstitute a vial of the lyophilized fluorescent, amine-reactive LIVE/DEAD^TM^ dye with DMSO in 500 µL (instead of the 50 µL volume recommended by the manufacturer). Add 0.2 μL of the reconstituted LIVE/DEAD^TM^ dye to 50 μL of MACS buffer per sample (1:250).
*Note: You can store the remaining reconstituted LIVE/DEAD^TM^ dye in aliquots at -20 °C.*
  10. Resuspend cell pellet with 50 μL of LIVE/DEAD^TM^ dye containing MACS buffer and incubate at 4 °C for 10 min, protected from light.
  11. Prepare the staining solution by mixing the following antibodies in 50 µL of MACS buffer per sample: BV785-CD8 (diluted 1:200), APC-CD62L (1:400), PE/Cy7-CD45RA (1:400), FITC-CD95 (1:400), BV421-PD1 (1:50), and PE/Dazzle594-TIGIT (1:100). (CD62L, CD45RA and CD95 are markers for T_SCM_ cells, while PD1 and TIGIT serve as markers for exhausted T cells; see Figure 3A and B.)
  12. Wash the cells once with MACS buffer and resuspend in 50 μL of staining solution.
  13. Stain cells with the staining solution for 15 min at 4 °C, protected from light.
  14. Wash cells once with 200 μL of MACS buffer and resuspend in 100 µL of MACS buffer prior to flow cytometric analysis (Figure 3). Keep the cells on ice.
  15. Flow cytometry analysis: Acquire at least 1 × 10^4^ to 2 × 10^4^ events (live cells) by first gating on lymphocytes (using the forward and side scatters FSC-A/SSC-A), then gating on singlet cells (by excluding doublets, clumps, and debris using FSC-H/FSC-W and SSC-H/SSC-W), and then gating on live cells (by excluding dead cells based on the LIVE/DEAD^TM^ stain signal). For data analysis, first pre-gate on CD8**^+^** cells and then analyze the other T-cell subsets (see Figure 3).
**CRITICAL:** Be sure to include a positive control for the antibody staining and the setting of the flow cytometry parameters. We use activated T cells from healthy donors (stimulated two days before flow analysis), and follow the procedure described in step F1 and F2. For clinical applications, we recommend that you characterize your T cells for the expression of additional T-cell exhaustion markers (TIM-3, LAG-3, and CD39) (see Suppl. Figure S5 in Li et al. [2]).
- **Prepare RNP complexes**
  1. Generate gRNA complexes by mixing 20 μL of a given crRNA (100 pmol) with 20 μL of tracrRNA (100 pmol) at a molarity ratio of 1:1 in a PCR tube.
  2. Heat the mixture at 95 °C for 5 min.
  3. Cool to room temperature (15–25 °C) on the benchtop for at least 10 min.
  4. Store gRNA complexes at -20 °C for up to 1 year.
  5. Right before electroporation, generate RNP complexes by mixing 2 μL of gRNA complex (100 pmol) and 0.75 μL of Cas9 (~50 pmol) at a molarity ratio of 2:1 in a PCR tube per 5 × 10^5^ activated human T cells and incubate the mixture at room temperature for 10–20 min on the benchtop.
*Note: We use two gRNAs for gene repair in primary T cells because this increases the gene repair efficiency (Li et al. [2] Suppl. Table S1).*
  6. Mix the two RNP complexes immediately before electroporation.
- **Production and purification of adeno-associated virus (AAV) donor vectors**
  Transfect, purify, concentrate, and calculate the copy number of rAAV6 donor particles according to our previous protocol [30]. **See General note 2.**
- **Genome editing of human T cells and phenotyping of edited T cells by flow cytometry**
  The general workflow for gene editing of expanded patient-derived T cells is shown in Figure 4.
  **Figure 4.**
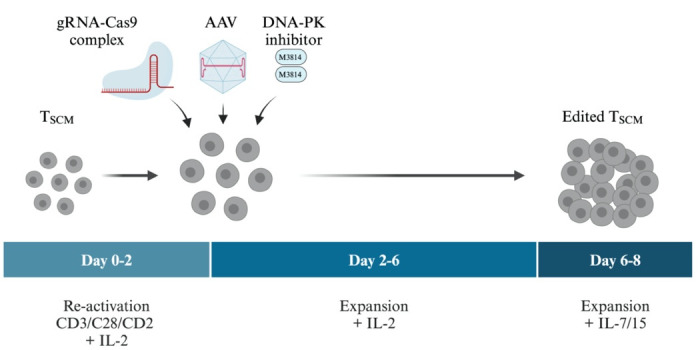
Workflow for CRISPR/Cas9 gene repair in expanded CD8^+^ T memory stem cells (T_SCM_) from patients with familial hemophagocytic lymphohistiocytosis (FHL). Starting from aliquots of expanded, patient-derived frozen T_SCM_-like cells, T cells are reactivated and expanded for 2 days with IL-2, then electroporated with RNPs, and immediately infected with recombinant adenovirus-associated virus (AAV) serotype 6 (carrying the DNA donor repair templates) in the presence or absence of a small molecule NHEJ inhibitor [DNA-PK inhibitor M3814 (Nedisertib)]. Transfected, infected T cells are first expanded for 4 days with IL-2 and then for two more days in the presence of IL-7 and IL-15.
  1. Thaw frozen vials from step C7 and culture T cells in ImmunoCult^TM^-XF T cell expansion medium with human IL-2 (25 ng/mL) in the presence of ImmunoCult^TM^ human CD3/CD28/CD2 T-cell activator (25 μL/mL) at a density of 1 × 10^6^/mL.
  2. Forty-eight hours post reactivation, harvest expanded T cells and count the cells using a hemacytometer.
  3. Prewarm ImmunoCult^TM^-XF T cell expansion medium (at least 1 mL per sample).
  4. Transfer 5 × 10^5^ T cells to a 1.5 mL Eppendorf tube and spin down at 300× *g* for 5 min at room temperature.
*Note: We generally electroporate 5 × 10^5^ to 10^6^ activated human T cells per electroporation reaction. The minimum cell number of T cells per electroporation is 3 × 10^5^.*
  5. Remove the supernatant and wash the cells once at room temperature with 1 mL of PBS.
  6. Pellet cells at 300× *g* for 5 min at room temperature and carefully remove the supernatant without disturbing the cell pellet.
  7. Suspend cells in 20 μL of P3 buffer (see Recipes) by gently pipetting up and down 10 times.
  8. Add freshly prepared RNPs (see steps D5 and D6) to the cell suspension and mix well by pipetting up and down 10 times.
  9. Transfer the mixture to a well of a 16-well nucleocuvette strip.
  10. Electroporate the cells using the EH-100 program of Lonza 4D-Nucleofector.
  11. After electroporation, add 75 μL of prewarmed ImmunoCult^TM^-XF T cell expansion medium containing human IL-2 (25 ng/mL) per well and gently mix cells.
  12. Transfer resuspended cells to an Eppendorf tube containing 500 µL of prewarmed culture medium.
  13. Transfer 1/5 to 2/5 of the cells in 200 μL of prewarmed ImmunoCult^TM^-XF T cell expansion medium containing human IL-2 (25 ng/mL) to a well of a 96-U bottom plate (corresponding to an estimated 1 × 10^5^ to 2 × 10^5^ cells based on the input).
  14. Add rAAV6 donor particles (from Section E) to the well containing the electroporated cells at a MOI of 1 × 10^6^ to 2 × 10^6^ genome copy/cell.
  15. Add 4 µL of M3814 (Nedisertib) inhibitor from a 50× working stock solution (250 µM; see Recipes) to a final concentration of 5 μM.
  16. After approximately 16 h, gently mix cells by pipetting up and down 10 times and pellet cells at 300× *g* for 5 min at 4 °C; then, carefully remove the supernatant without disturbing the cell pellet.
  17. Add 200 µL of prewarmed ImmunoCult^TM^-XF T cell expansion medium containing human IL-2 (25 ng/mL) and culture cells in a 96-well U bottom plate.
  18. After approximately 24 h, transfer cells to a 48-well plate and culture cells in 350–400 μL of medium.
*Note: To achieve sufficient expansion of edited human T cells, we recommend gradually expanding cells from a 96-well U-bottom plate to a bigger plate (48-well plate, 24-well plate, 12-well plate, and 6-well plate) and monitoring the culture every day until the cells are used for downstream analysis or application.*
  19. Four days post-infection, change the medium to fresh ImmunoCult^TM^-XF T cell expansion medium with human IL-7 (5 ng/mL) and IL-15 (5 ng/mL) for another two days (1.5 mL for a 12-well plate or 3 mL for a 6-well plate).
  20. Analyze the cells by flow cytometry (stain cells as described in steps C9–C15) (see Figure 5).
    **Figure 5.**
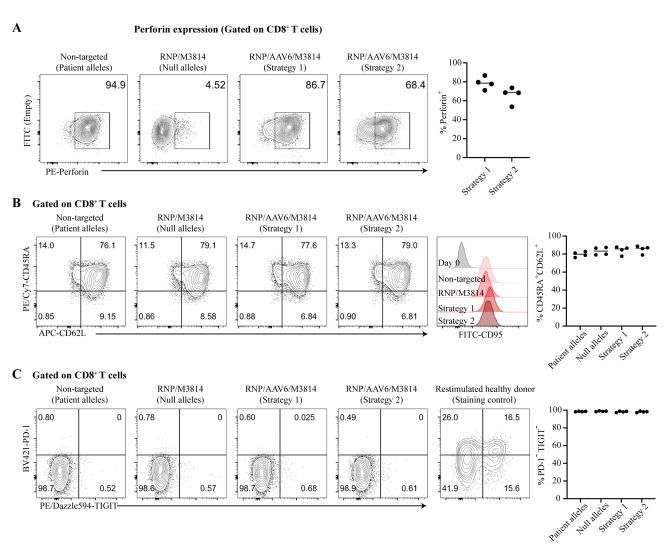
Efficient repair of perforin mutations CD8^+^ T memory stem cells (T_SCM_) from a patient with familial hemophagocytic lymphohistiocytosis (FHL). A. The efficiency of *PRF1* gene repair was assessed by flow cytometry analysis of intracellular perforin expression in CD8^+^ T cells from a patient with FHL. T cells were reactivated two days before the analysis, then stimulated with PMA and ionomycin for 6 h in the presence of brefeldin A, before fixation, permeabilization, and staining for precursor and mature forms of perforin with monoclonal antibody B-D48 [31]. Left: Representative flow cytometry plots after the indicated treatments. An inactive precursor form of perforin is detectable in the CD8^+^ T cells of this patient (patient alleles) because the patient had a c.1349C>T(T450M) missense mutation, known to impair the proteolytic maturation of perforin [32]. By contrast, perforin staining is almost absent in CD8^+^ T cells that received RNP without AAV6 (null alleles, generated by NHEJ events causing frameshift mutations). Right: Frequency of perforin-expressing CD8^+^ T cells after perforin repair by RNP/AAV6/M3814 treatment. Each dot represents one independent experiment, horizontal lines indicate medians. [Note: In these experiments, we also used human BJAB lymphoma cells and a CD19-CD3 bispecific antibody [33] to activate the patient’s T cells, but we later found (using T cells from healthy donors) that this additional activation mode is not necessary (Li et al. [2], Figure 5D)]. B. Left: Representative phenotypes by flow cytometry of repaired patient-derived CD8^+^ T cells after the indicated treatments. CD45RA^+^CD62L^+^ T cells were almost exclusively CD95^+^, differentiating them from naïve T cells and identifying them as T_SCM_-like. Right: Frequency of CD45RA^+^CD62L^+^CD8^+^ T cells after the indicated treatments. Each dot represents one independent experiment, horizontal lines indicate medians. C. Repaired CD8^+^ T cells did not overexpress the T-cell exhaustion markers PD-1 and TIGIT. Other T-cell exhaustion markers (TIM-3, LAG-3, and CD39) were also tested and found not to be overexpressed (see Suppl. Figure S5 in Li et al. [2]). Frequency of PD1-TIGIT- CD8^+^ T cells after the indicated treatments. Each dot represents one independent experiment, horizontal lines indicate medians. RNP, ribonucleoprotein complexes of gRNA and Cas9 nuclease; AAV6, adeno-associated virus serotype 6 providing the DNA repair template; M3814, DNA-PK inhibitor M3814 (Nedisertib). Non-targeted cells correspond to untransfected patient cells. Reprinted/adapted from Figure 6 in Li et al. [2].
- **Assessment of gene-targeting efficiency by intracellular perforin staining**
  1. Reactivate 5 × 10^5^ to 1 × 10^6^ edited T cells per well of a 24-well plate with ImmunoCult^TM^-XF T cell expansion medium with human IL-2 (25 ng/mL) in the presence of ImmunoCult^TM^ human CD3/CD28/CD2 T-cell activator (25 μL/mL) at a density of 1 × 10^6^/mL for two days.
  2. Stimulate 1 × 10^5^ to 2 × 10^5^ reactivated T cells with PMA (50 ng/mL) and ionomycin (500 ng/mL) for 6 h in the presence of 1× Brefeldin A solution in a 96-well U bottom plate.
  3. Pipette several times and pellet cells at 300× *g* for 5 min at 4 °C in the same 96-well U-bottom plate. Then, carefully remove the supernatant without disturbing the cell pellet.
  4. Wash the cells once with 100 μL of MACS buffer and pellet cells at 300× *g* for 5 min at 4 °C. Carefully remove the supernatant without disturbing the cell pellet.
  5. Add 0.2 μL of the reconstituted LIVE/DEAD^TM^ dye per 50 μL of MACS buffer.
  6. Resuspend cell pellet with 50 μL of LIVE/DEAD^TM^ dye containing MACS buffer and incubate at 4 °C for 10 min, protected from light.
  7. Wash the cells once with MACS buffer and resuspend in 50 μL of MACS buffer.
  8. Stain for surface markers as described in step C11 and incubate for 15 min on ice, protected from light.
  9. Wash the cells once with MACS buffer.
  10. Thoroughly resuspend cells in 100 μL of Cytofix/Cytoperm^TM^ solution for 20 min at 4 °C in the dark.
  11. Prepare the perforin staining solution by diluting 1 µL of anti-perforin antibody in 50 µL of 1× Perm/Wash^TM^ solution.
*Note: We use the monoclonal antibody B-D48 that detects both precursor and mature forms of perforin (Hersperger et al. [31]; see Li et al. [2] Figure 6D).*
  12. Wash cells two times in 1× Perm/Wash^TM^ solution, pellet, and remove supernatant.
  13. Thoroughly resuspend fixed/permeabilized cells in 50 μL of 1× Perm/Wash^TM^ solution containing anti-perforin antibody. Incubate at 4 °C for 30 min in the dark.
  14. Wash cells two times with 200 μL of 1× Perm/Wash^TM^ solution and resuspend in 100 μL of MACS buffer prior to flow cytometry analysis (Figure 5).
- **Assessment of knock-in efficiencies by on-target PCR, cloning, and sequencing of *PRF1* alleles**
*Note: This step serves to confirm the correct integration site and to quantify the fractions of HDR and NHEJ events. Alternatively to the cloning procedure below, you could do PacBio long-read amplicon sequencing.*
  1. Extract genomic DNA from 5 × 10^5^ to 1 × 10^6^ targeted bulk T cells with 500 µL of QuickExtract^TM^ DNA extraction solution (at a density of 1 × 10^6^ to 2 × 10^6^ cells/mL).
  2. Mix well and briefly vortex to completely lyse cells. Transfer 50–100 µL of lysed cells to a PCR tube.
  3. Denature the genomic DNA using a PCR machine and the following temperature cycle: 65 °C for 15 minutes, 68 °C for 15 min, 95 °C for 15 min, and 4 °C. The denatured genomic DNA is ready for PCR amplification.
  4. Amplify the targeted site using PrimeSTAR^®^ GXL DNA polymerase with gene-specific primers that bind outside of the homology arms (HA) (*PRF1*.5HA-Fw and *PRF1*.3HA-Rv; Li et al. [2] Suppl. Table S8). PCR conditions: 30 cycles (98 °C for 10 s, 60 °C for 15 s, 68 °C for 1 min per kb) in 25 µL containing 5 µL of 5× PrimeSTAR^®^ GXL buffer, 0.5 µL of PrimeSTAR^®^ GXL polymerase, 2 µL of NTP mixture (2.5 mM each), plasmid template 1 µL (2.5–250 ng), primers 1 µL each (10 µM), filled up to 25 µL with water.
  5. Clone the purified PCR products into sequencing vector pJET1.2/Blunt (CloneJET PCR cloning kit).
  6. Pick approximately 100 colonies into 96-well microtiter plates (MTP) (pre-filled with 200 µL of LB agar containing 100 µg of ampicillin), incubate overnight at 37 °C, and send the MTP with suitable primers to MTP sequencing (Sanger Sequencing Services Premium Run, LGC Biosearch Technologies).
  7. Open sequence files one by one with ApE software [34] and count nontargeted, HDR, and NHEJ events (Table 2 and Suppl. Figure S8).
*Note: Using two sgRNAs and NHEJ inhibitor M3814, we observed median allele editing frequencies of approximately 60% with strategy 1 and 40% with strategy 2 (n ≥ 3; see also Li et al. [2] Figure 6B and Suppl. Table S2).*
    **Table 2.** Knock-in efficiencies measured by sequencing of targeted alleles. Percentage of HDR, NHEJ, and non-targeted events were calculated as illustrated in Figure S8.

	Strategy 1 (n = 4)				Strategy 2 (n = 3)		
% HDR	61.8	73.0	61.5	61.7	38.2	63.3	41.1
% NHEJ	35.3	27.0	35.9	36.2	58.8	23.3	58.9
% Non-targeted	2.9	0	2.6	2.1	2.9	13.3	0
- **Genome-wide identification of off-target sites with GUIDE-seq**
  For *Tn5*-modified GUIDE-seq experiments and data analysis [35], please follow our previous *Bio-protocol* (Tran et al. [36]; see Section H. *Tn5* transposase-mediated GUIDE-seq). **See General note 3.**

## Data analysis

Genome editing efficiency of gRNAs: See Figure S6 for an example of an “inference of CRISPR edits” (ICE) analysis. Flow cytometry data are analyzed with the commercial software FlowJo. Basic expertise in flow cytometry is required to assess the phenotype of patient T cells before and after gene repair and to quantitatively assess cell yields of edited T cells and the gene repair efficiency by measuring the percentage of repaired T cells expressing perforin in response to T-cell activation.

Phenotyping of T cells: After 10 days of culture with IL-7 and IL-15, more than 90% of CD8^+^ T cells were T_SCM_ cells without signs of T-cell exhaustion; after gene editing, >75% of the T cells retained this phenotype (Figure 3, Figure 5; see also Li et al. [2] Suppl. Figure S5).

Yield of edited cells: Without NHEJ inhibitor M3814, T cells from healthy donors yielded 8 × 10^5^ to 9 × 10^5^ edited T cells after 6 days (starting from 10^5^ T cells) (see Li et al. [2] Figure 5C). The addition of M3814 led to a 30%–40% reduction in cell yield. **See General note 4.** Using two gRNAs and NHEJ inhibitor M3814, we observed median gene editing efficiencies of approximately 80% and 70% with strategy 1 and strategy 2, respectively (n = 4; see also Li et al. [2] Figure 6B and Suppl. Table S2).

Knock-in efficiencies by sequencing and percentage of HDR, NHEJ, and non-targeted alleles: These data are given in Table 2. Please see Figure S8 in this *Bio-Protocol* for how to identify HDR, NHEJ, and non-targeted alleles by sequencing and how to calculate the contribution of each to the total number of alleles after gene editing. We recommend sequencing a minimum of 25–30 alleles to obtain representative results. To make sure gene targeting efficiencies are reproducible, experiments should be repeated at least two times.

## Validation of protocol

Validation experiments for both gene editing strategies (Figure 1) are shown in our article Li et al. [2], as follows: *PRF1* gene editing conditions were optimized using T cells from healthy donors [Figure 5 and Table S1; one independent experiment per donor (n = 3)]. Efficient repair of perforin mutations in T cells from a patient with FHL2 was demonstrated in Figure 6, Table S1, and Suppl. Figure S4 [four independent experiments (n = 4)]. Patient T cells showed a T_SCM_-like phenotype after gene repair [Figure 6D; four independent experiments (n = 4)] and did not show increased levels of the T-cell exhaustion markers PD-1, TIGIT, TIM-3, LAG-3, and CD39 [Figure 6E and Suppl. Figure S5; four independent experiments (n = 4)].

## General notes and troubleshooting

**General notes**

1. Because gRNAs can vary widely in gene editing efficiency, it is important to test this parameter experimentally. There are different methods available (see Tran et al. [36] steps A1–A4). For example, gRNAs can be tested in vitro using synthetic crRNAs (or in vitro–transcribed gRNAs), Cas9 protein, and PCR amplicons. gRNAs can also be tested in cell lines or in primary cells, which is considered more reliable than in vitro testing. Popular assays for quantitative assessment of the efficiency of target site cleavage are the Surveyor/T7 endonuclease I (T7EI) assay [22] or Sanger sequencing followed by ICE analysis [24] or TIDE analysis [37]. Commercial kits for testing gRNA efficiency are available as well, such as the GeneArt^TM ^Genomic Cleavage Detection Kit (Invitrogen), the Alt-R^TM ^Genome Editing Detection Kit (IDT), or the Guide-it^TM ^Complete sgRNA Screening System (Takara Bio, Inc.).
2. An alternative to AAV production that may be less costly and less complex to scale up is non-viral T-cell engineering using CRISPR-Cas9 and virus-free single-stranded DNA as a template donor, as recently demonstrated for IL-7-IL-15-expanded, blood-derived T_SCM_ at a clinical scale [8]. Another advantage of single-stranded DNA template donors is that they can be synthesized to approximately 8,000 nucleotides in length, enabling the use of longer repair templates than AAV, which has only a packaging capacity of 4–4.4 kb.
3. For clinical applications, it is necessary to establish safety profiles for the gRNAs used and to carefully assess potential genome-wide off-target sites [38,39]. To this end, we have successfully used *Tn5* transposase-mediated GUIDE-seq (see Li et al. [2]; Tran et al. [35]). To improve the fidelity of gene repair, an engineered high-fidelity Cas9 such as Alt-R^TM^ *S.p.* HiFi Cas9 Nuclease V3 from IDT should be used [40].
4. For scale-up and good manufacturing practice (GMP)-compliant experiments, we suggest following the procedure described by Shy et al. [8] (Figure 5C). Shy et al. [8] expanded electroporated T_SCM_ in G-Rex 100 M gas-permeable culture vessels (Wilson Wolf Manufacturing) in media supplemented with 100 U/mL of IL-7 and 10 U/mL IL-15 every 2–3 days for up to 10 days.

**Troubleshooting**

**Table:** 

Problem	Potential solution
Failed ICE analysis (step A4)	Try to improve the PCR, the sequencing quality, or both. A clearly defined PCR band and high-quality sequencing data are required for successful ICE analysis.
Low gene knock-out efficiency (step A4)	Efficient gRNAs are required: Try different gRNAs. Please test several gRNAs to identify highly efficient ones.
Inefficient AAV production/low AAV titers (section E)	Use single-stranded DNA as a donor template if the size of the donor template is close to 4.4 kb.
Inefficient expansion of T cells after gene editing (step F18/F19)	Expand cells gradually from a 96-well U-bottom plate to a bigger plate (48-, 24-, 12-, and 6-well plates) and monitor the culture every day until cells have sufficiently expanded to be used in downstream analysis or applications.

## Supplementary information

The following supporting information can be downloaded here:

1. Figure S1. Two-part gRNA hybridized to form a crRNA:tracr RNA complex.
2. Figure S2. gRNA design with free CrispRGold software(Graf et al., 2019).
3. Figure S3. gRNA design with free CrispRGold software(Graf et al., 2019).
4. Figure S4. Output of CrispRGold(Graf et al., 2019) gRNA design request with off-target sites shown in expanded mode.
5. Figure S5. Cloning oligo nucleotides with BbsI sticky ends for gRNA standard vectors(pX330, MSCV retroviral vectors, etc.).
6. Figure S6. Analysis of the genome editing efficiency of the guide RNA PRF1.2 with the free web-based tool“inference of CRISPR edits”(ICE)(Conant et al., 2022).
7. Figure S7. Plasmid map of the pAAV-GFP control vector.
8. Figure S8. Example of HDR, NHEJ, and WT events after gene repair as determined by Sanger sequencing.
